# Neonatal varicella complicated by Staphylococcus aureus lung abscess in a preterm infant: a case report

**DOI:** 10.3389/fped.2026.1845229

**Published:** 2026-07-06

**Authors:** Lingxia Zhao, Yuqiong Ming, Lingkong Zeng, Minghui Yi, Xuwei Tao, Wenhao Yuan

**Affiliations:** 1 Department of Neonatology, Wuhan Children’s Hospital (Wuhan Maternal and Child Healthcare Hospital), Tongji Medical College, Huazhong University of Science and Technology, Wuhan, Hubei, China; 2 Department of Radiology, Wuhan Children’s Hospital (Wuhan Maternal and Child Healthcare Hospital), Tongji Medical College, Huazhong University of Science and Technology, Wuhan, Hubei, China

**Keywords:** case report, lung abscess, metagenomic next-generation sequencing (mNGS), neonatal varicella, preterm infant, *Staphylococcus aureus*

## Abstract

**Background:**

Neonatal varicella is a rare but potentially life-threatening condition, particularly in preterm infants. Although secondary bacterial infections are common complications, deep organ involvement such as lung abscess formation is exceedingly rare. Reports describing neonatal varicella complicated by Staphylococcus aureus lung abscess are scarce.

**Case presentation:**

We report a 26-day-old preterm infant (32 weeks' gestation, birth weight 1.94 kg) who developed a progressive vesiculopustular skin lesions and respiratory deterioration following exposure to maternal varicella. Despite initial topical treatment, the rash rapidly worsened and was accompanied by poor responsiveness, apnea, cyanosis, and hypothermia. On admission, the infant presented with extensive skin lesions, respiratory distress requiring non-invasive ventilation, coagulopathy, and thrombocytopenia. Intravenous acyclovir and immunoglobulin were initiated. Although the skin lesions gradually crusted and resolved, respiratory abnormalities persisted and oxygen supplementation remained necessary. Chest imaging subsequently revealed a right upper lobe abscess. Blood cultures remained negative; however, respiratory metagenomic next-generation sequencing (mNGS) identified *Staphylococcus aureus,* confirming secondary bacterial infection. Initial antibiotic therapy with vancomycin was selected because of severe pulmonary infection and the local prevalence of oxacillin-resistant Staphylococcus aureus. However, subtherapeutic trough concentrations and limited clinical response prompted a switch to linezolid. Following treatment adjustment, the infant showed gradual clinical and radiographic improvement and was discharged in stable condition. Follow-up imaging confirmed complete resolution of the lung abscess.

**Conclusions:**

This case represents a rare presentation of neonatal varicella complicated by *Staphylococcus aureus* lung abscess in a preterm infant. It highlights that apparent resolution of cutaneous lesions does not exclude ongoing deep-seated bacterial infection and that persistent respiratory abnormalities should prompt early imaging evaluation, even in the absence of typical respiratory signs. In culture-negative cases, mNGS may facilitate pathogen identification and guide targeted antimicrobial therapy. Early recognition, individualized antimicrobial management, and therapeutic drug monitoring are important for optimizing outcomes in high-risk neonates. This case also underscores the importance of timely post-exposure prophylaxis and the limited availability of varicella-zoster immune globulin (VZIG) in some regions.

## Background

Varicella is a vaccine-preventable childhood disease caused by varicella-zoster virus (VZV). Although typically a mild and self-limited illness, it can lead to severe complications in infants and immunocompromised individuals (1). While the highest incidence of varicella in China occurs among preschool children aged 1–6 years, infants under 1 year of age are at a significantly increased risk of severe disease (2). Neonatal varicella is rare but potentially life-threatening, with clinical manifestations ranging from localized cutaneous vesicles to disseminated infection involving the lungs, liver, and central nervous system (3). Without timely intervention, severe cases may result in mortality rates of up to 30% (4).

The severity of neonatal varicella is largely determined by the timing of maternal infection. The highest risk occurs when maternal onset develops between 5 days before and 2 days after delivery, a period during which adequate transplacental transfer of protective antibodies is not achieved (5). In addition to perinatal transmission, postnatal horizontal transmission via respiratory droplets or direct contact may also occur. Preterm and low-birth-weight infants are particularly vulnerable due to their immature immune systems and reduced maternal antibody acquisition (6).

Despite the implementation of vaccination programs, susceptibility among women of childbearing age remains a concern. A recent epidemiological study in Beijing (2014–2023) reported single-dose and two-dose varicella vaccination coverage of approximately 85% and 60%, respectively, indicating that a proportion of women remain without protective immunity (2). In contrast, countries with universal infant vaccination programs, such as the United States, Canada, and Australia, have reported marked reductions in the incidence of both congenital and neonatal varicella (6).

Secondary bacterial infections are well-recognized complications of varicella, most commonly involving the skin and soft tissues, with *Staphylococcus aureus* and Group A Streptococcus as the predominant pathogens (1, 7). However, visceral involvement such as pneumonia is rare, and lung abscess formation following varicella is exceptionally uncommon, with only isolated cases reported in the literature (5).

These gaps in prevention and recognition underscore the need for heightened clinical awareness of atypical and severe presentations of neonatal varicella. Here, we report a rare case of a preterm infant who developed a *Staphylococcus aureus* lung abscess following late-onset postnatal varicella infection. This case highlights the importance of maintaining a high index of suspicion for secondary bacterial complications, even after apparent resolution of cutaneous lesions. It also underscores the value of early pathogen identification and individualized antimicrobial therapy in the management of complex neonatal infections. To our knowledge, lung abscess following neonatal varicella in a premature infant has rarely been reported.

## Case presentation

A 26-day-old male infant was admitted to Wuhan Children's Hospital on December 19, 2025, with a two-day history of a progressive rash.

He was the third child, born via vaginal delivery at 32 weeks and 5 days of gestation, with a birth weight of 1.94 kg. Due to prematurity, he was hospitalized locally for the first 10 days of life. After discharge on day 10, he had close contact with his mother. On day 13 of life, his mother developed pustular lesions on her trunk, which were clinically diagnosed as varicella. She had no prior history of varicella infection and had not received varicella vaccination.

The infant remained asymptomatic until day 24 of life, when erythematous maculopapules appeared on the face. The rash rapidly spread to the scalp, neck, and trunk, with relative sparing of the extremities, and evolved into vesiculopustular lesions with partial ulceration. Based on the exposure history, the incubation period was estimated to be approximately 14 days.

Despite two days of topical treatment with iodophor and erythromycin ointment, the rash continued to worsen and was accompanied by poor responsiveness, decreased feeding, apnea, cyanosis, and hypothermia, prompting hospital admission. At presentation, the skin lesions were considered consistent with varicella infection, without clinical evidence of secondary bacterial skin infection such as cellulitis, purulent drainage, or abscess formation. Therefore, systemic antibacterial therapy was not initiated initially and treatment was focused on antiviral therapy and supportive care.

**Figure 1:**
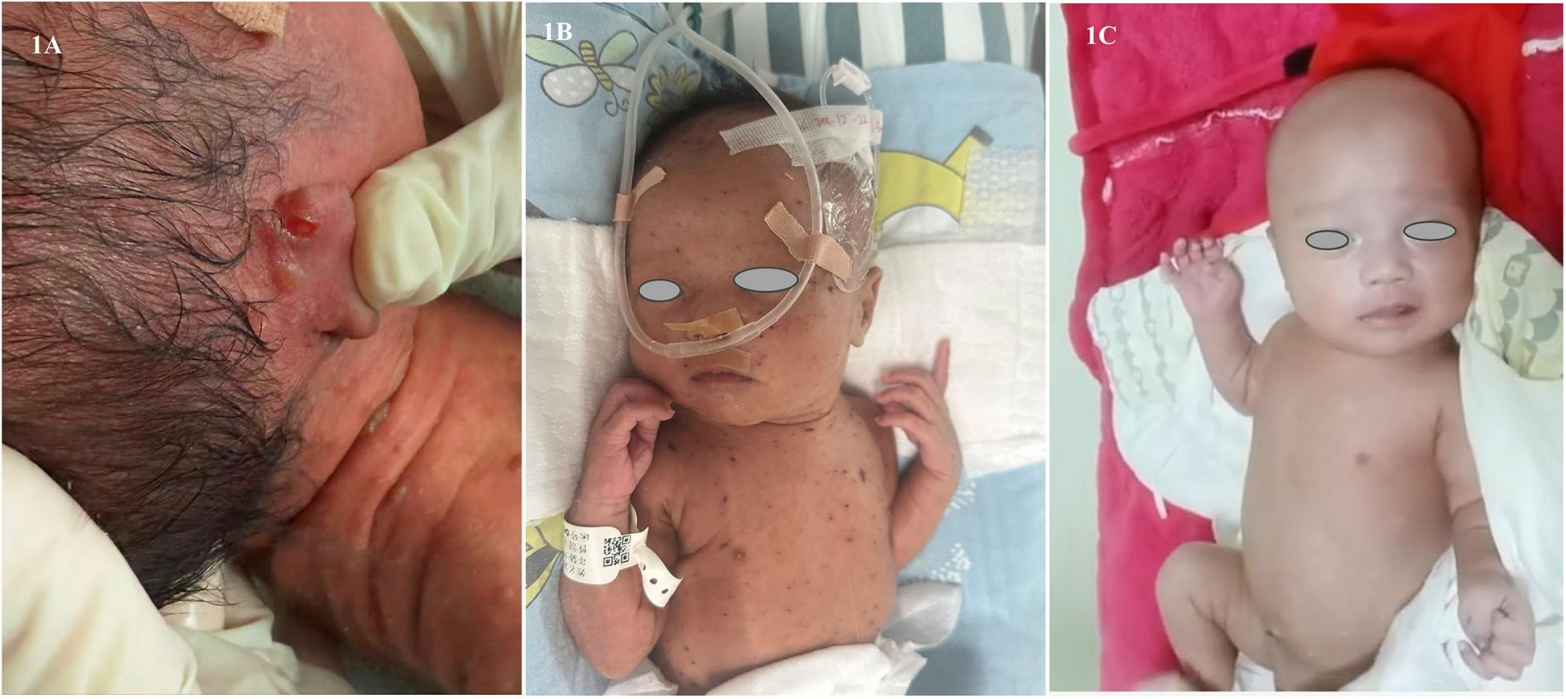
Evolution of cutaneous lesions in a preterm infant with neonatal varicella. **(A)** Early-stage vesiculopustular rash involving the face and trunk. **(B)** Progressive lesion development after hospital admission with increased density and partial ulceration. **(C)** Complete resolution of skin lesions at follow-up without residual scarring.

On admission, the infant appeared lethargic, with a body temperature of 35.5 °C. Physical examination revealed dense maculopapular and vesiculopustular lesions with areas of ulceration involving the head, face, trunk, neck, and groin, with relative sparing of the extremities (Figures 1A,B). Circumoral cyanosis was noted, along with shallow and irregular respiration and frequent apneic episodes. Breath sounds were coarse without audible rales. Cardiac and abdominal examinations were unremarkable. To evaluate for possible systemic involvement, echocardiography and abdominal ultrasonography were performed and showed no significant abnormalities, with no evidence of endocarditis, hepatosplenomegaly, intra-abdominal infection, or other organ involvement.

## Clinical course

### Hospital days 1–3 (illness days 3–5): antiviral therapy and initial deterioration

Upon admission, intravenous acyclovir (10 mg/kg every 8 h for 10 days) and intravenous immunoglobulin (1 g/kg for 2 days) were initiated. Given the presence of hypothermia, coagulopathy, and thrombocytopenia, the infant was considered at high risk for disseminated intravascular coagulation (DIC) and received plasma transfusion (20 mL/kg). Non-invasive ventilation and nasogastric feeding were required due to apnea and hypoxemia.

**Figure 2:**
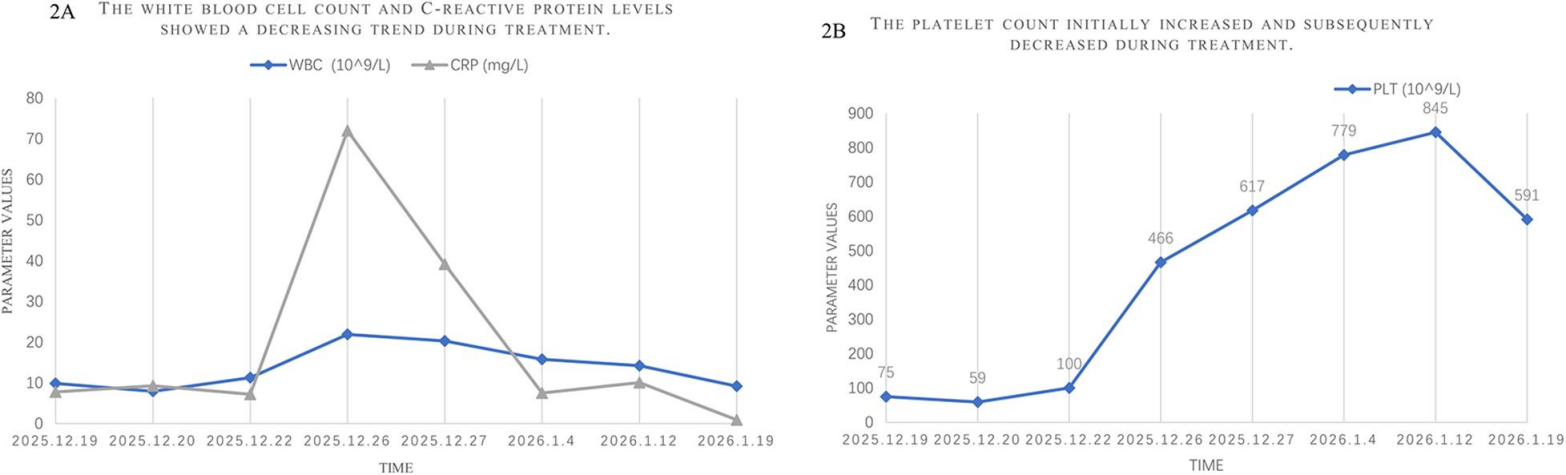
Dynamic changes in laboratory parameters during hospitalization and follow- up. **(A)** Trends in white blood cell (WBC) count and C-reactive protein (CRP) levels over the disease course. **(B)** Platelet count dynamics showing initial thrombocytopenia followed by reactive thrombocytosis during recovery.

Initial laboratory investigations demonstrated thrombocytopenia and elevated inflammatory markers. On illness day 2, chest radiography showed increased lung markings. Complete blood count revealed a platelet count of 80 × 10⁹/L and a C-reactive protein (CRP) level of 12.8 mg/L. On illness day 3, coagulation studies showed prolonged APTT (86.5 s), PT of 14.6 s, D-dimer of 1.79 mg/L, and fibrinogen of 1.23 g/L. Platelet count decreased to 75 × 10⁹/L, CRP was 7.75 mg/L, and procalcitonin was 0.170 ng/mL. Immunological testing showed reduced IgG (4.42 g/L) and complement C3 (0.43 g/L). Trends in platelet count and CRP are illustrated in Figure 2. Liver and renal function tests, myocardial enzymes, and electrolytes were within normal ranges. Metabolic screening, including blood amino acids and urine organic acids, was unremarkable. Trends in inflammatory markers and platelet counts during hospitalization and follow-up are illustrated in Figures 2A,B.

### Hospital days 4–11 (illness days 6–13): progression to pneumonia and lung abscess

On illness day 6, blood metagenomic next-generation sequencing (mNGS) detected varicella-zoster virus (VZV) with 855,695 sequence reads, which was subsequently confirmed by PCR.

**Figure 3:**
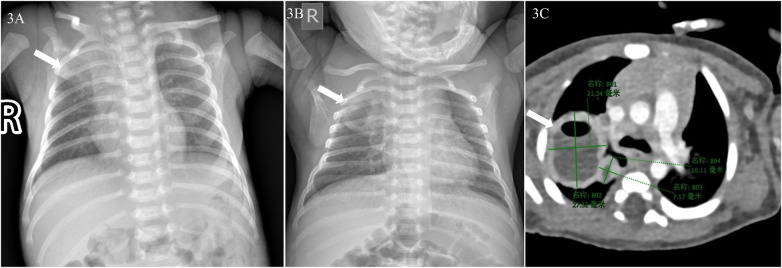
Multimodal imaging findings of lung abscess secondary to neonatal varicella. **(A)** Chest x-ray on illness day 6 (hospital day 4) showing right upper lobe consolidation (arrow). **(B)** Chest x-ray on illness day 9 (hospital day 6) demonstrating progression with suspected abscess formation (arrow). Characterization phase: **(C)** Coronal multiplanar reconstruction (MPR) CT image showing a right upper lobe abscess measuring 21 × 27 × 23 mm (arrow).

Despite complete crusting of the skin lesions by illness day 8, the infant remained tachypneic and oxygen-dependent. Chest radiography at this stage revealed patchy consolidation in the right upper lobe (Figure 3A), prompting initiation of amoxicillin–clavulanate. However, repeat imaging on illness day 11 demonstrated disease progression with bilateral pneumonia and a suspected abscess in the right upper lobe (Figure 3B).

Laboratory findings showed a marked inflammatory response, with CRP increasing to 72.0 mg/L and white blood cell count to 21.91 × 10⁹/L. Blood cultures obtained during hospitalization remained negative. However, respiratory mNGS identified *Staphylococcus aureus* (17,835 reads; >1.0 × 10⁶ copies/mL), confirming secondary bacterial infection and providing microbiological evidence to guide antimicrobial therapy. In combination with the progression of pulmonary lesions and markedly elevated inflammatory markers, targeted anti-staphylococcal therapy was subsequently initiated. Serum galactomannan testing and T-SPOT.TB assay were negative, and lymphocyte subset analysis (TBNK) was within normal limits. Antibiotic therapy was escalated to intravenous vancomycin (15 mg/kg every 8 h).

### Hospital days 16–34 (illness day 18 to discharge): treatment adjustment and recovery

To further characterize the lesion, contrast-enhanced chest computed tomography (CT) was performed on illness day 16, which confirmed a right upper lobe abscess measuring 21 × 27 × 23 mm (Figure 3C, Supplementary Figure S1A). By this time, the cutaneous lesions had completely resolved. In addition, lung ultrasound (LUS) demonstrated irregular hypoechoic areas with indistinct margins and heterogeneous internal echotexture, while color Doppler imaging revealed intralesional vascular signals, supporting the diagnosis of an active inflammatory lesion (Supplementary Figures S1B,C).

Following initiation of vancomycin therapy, serial chest radiographs showed no significant improvement in the pulmonary lesion (Supplementary Figure S2A), which, together with subtherapeutic vancomycin trough levels (4.31 µg/mL), led to a switch of antibiotic treatment to intravenous linezolid (10 mg/kg every 8 h) on illness day 24.

During hospitalization, the infant developed oral candidiasis with an elevated (1,3)-β-D-glucan level (96.32 pg/mL), which was treated with fluconazole for 7 days. Reactive thrombocytosis was managed with dipyridamole. By illness day 27, β-D-glucan levels had normalized, and antifungal therapy was discontinued.

After switching to linezolid, by illness day 34, follow-up chest radiography demonstrated marked resolution of the lung abscess (Supplementary Figure S2B), accompanied by normalization of CRP levels. The infant was discharged on oral linezolid.

## Follow-up

**Figure 4:**
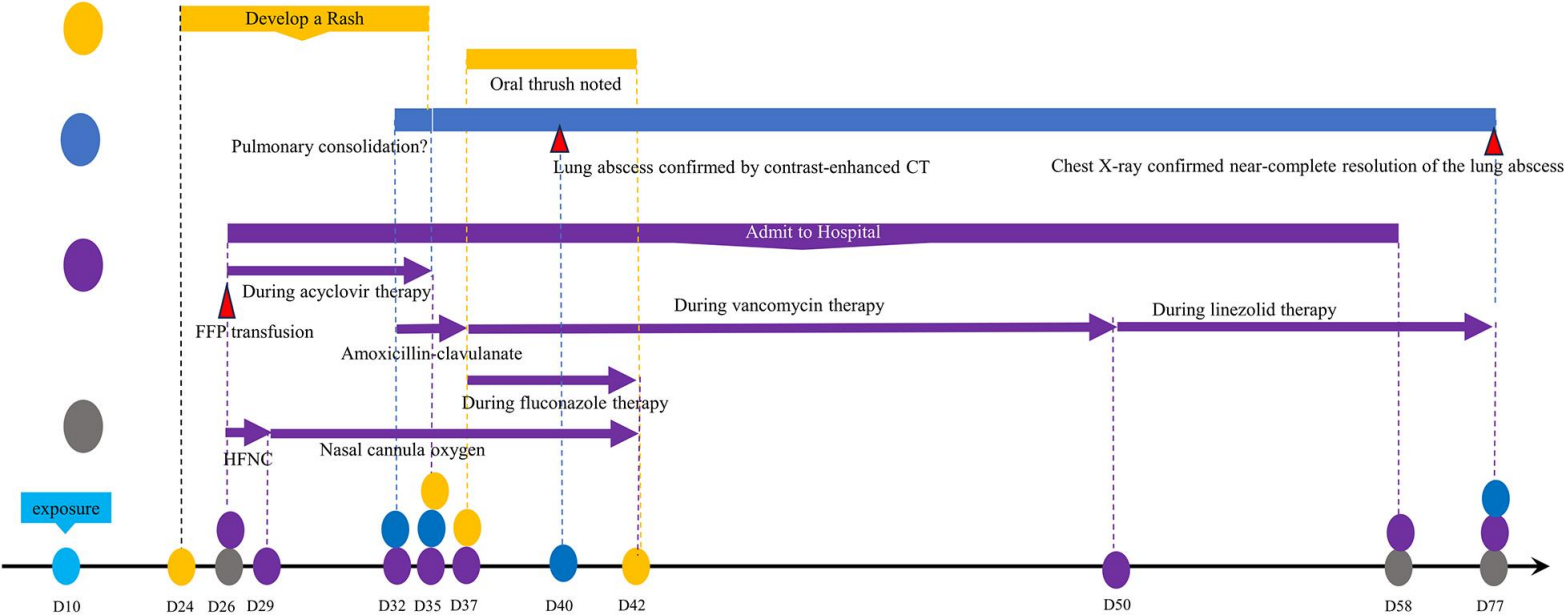
Clinical timeline of disease course and management. The timeline summarizes key clinical events from symptom onset to post-discharge follow-up. Hospital days are counted from admission (Day 1). Clinical features, diagnostic findings, and therapeutic interventions are presented in chronological order. FFP, fresh frozen plasma; HFNC, heated humidified high-flow nasal cannula.

At 18 days post-discharge, follow-up chest radiography showed complete resolution of the pulmonary lesion (Supplementary Figure S2C). Oral linezolid was discontinued after a total treatment duration of 4 weeks. Follow-up examination showed complete resolution of the skin lesions (Figure 1C). The infant remains under follow-up for retinopathy of prematurity (zone II, stage 1). The overall clinical course, including symptom progression, therapeutic interventions, and follow-up, is summarized in Figure 4.

## Discussion

We report a rare case of neonatal varicella complicated by a Staphylococcus aureus lung abscess in a preterm infant. While secondary bacterial superinfection is a well-recognized complication of varicella (1, 7), visceral involvement such as lung abscess formation is exceedingly uncommon, with only isolated cases reported in older infants (5). Other pulmonary complications, including empyema, have been described, most commonly caused by Group A Streptococcus and typically presenting with fever and respiratory symptoms (8, 9). This case expands the current clinical spectrum of neonatal varicella and underscores the importance of maintaining vigilance for deep-seated bacterial infections, even after apparent cutaneous improvement.

The severity of neonatal varicella is strongly influenced by the timing of maternal infection. As highlighted by Longbottom and Lyall (6), the highest risk of severe disease occurs when maternal rash develops between 5 days before and 2 days after delivery, due to insufficient transplacental transfer of protective antibodies. In the present case, maternal infection occurred on day 13 postpartum, and the infant developed symptoms 14 days later, consistent with postnatal horizontal transmission (6). The infant's clinical manifestations—including rapidly progressive rash, apnea, hypothermia, coagulopathy, and thrombocytopenia—were consistent with severe neonatal varicella. Prematurity likely contributed to disease severity, as transplacental antibody transfer predominantly occurs during the third trimester, resulting in reduced passive immunity in preterm infants (10, 11).

In addition, maternal susceptibility remains an important contributing factor. Although high seroprevalence of VZV antibodies has been reported in some populations (6), vaccination coverage and immunity among women of childbearing age remain suboptimal in certain regions. Yu et al. (2) reported that despite relatively high vaccination coverage in Beijing, a considerable proportion of women lack protective immunity. In our case, the mother's lack of prior infection or vaccination suggests immunological susceptibility, which, combined with the infant's prematurity, may have increased the risk of both severe varicella and subsequent bacterial complications.

The pathogenesis of lung abscess formation in this context is likely multifactorial, involving both local and systemic mechanisms. Disruption of the skin barrier by widespread vesiculopustular lesions may facilitate bacterial entry, while virus-induced immune dysregulation further predisposes to secondary infection. Previous studies have shown that VZV can impair phagocyte function and damage respiratory mucosa, thereby promoting bacterial invasion (5, 7). In this case, the temporal association between extensive skin involvement and subsequent pulmonary infection, together with the detection of S. aureus in respiratory specimens by mNGS, supports hematogenous dissemination as the most plausible mechanism, despite negative blood cultures.

This case also highlights an important diagnostic challenge. Although the cutaneous lesions began to resolve early in the disease course, the infant remained oxygen-dependent, which ultimately prompted further investigation and led to the diagnosis of lung abscess. This dissociation between apparent dermatological improvement and ongoing systemic disease represents a potential diagnostic pitfall. Clinicians should therefore maintain a low threshold for imaging in neonates with persistent respiratory symptoms following varicella, particularly in preterm infants.

The differential diagnosis included neonatal herpes simplex virus infection, bacterial sepsis with cutaneous manifestations, and other causes of neonatal vesiculopustular eruptions. However, the history of maternal varicella infection, the characteristic progression of skin lesions, and the detection of VZV by mNGS strongly supported the diagnosis of neonatal varicella. The subsequent identification of Staphylococcus aureus in respiratory specimens further confirmed secondary bacterial superinfection as the cause of the pulmonary complication.

The clinical presentation in this case was notably atypical. Unlike previously reported cases of varicella-associated pulmonary complications, which often present with fever, cough, and tachypnea (5), this infant exhibited minimal respiratory symptoms apart from oxygen dependence and apnea. This atypical presentation likely reflects the immature immune response and blunted inflammatory manifestations characteristic of preterm neonates, further complicating timely diagnosis.

Management of this case posed several therapeutic challenges. Initial treatment with vancomycin was guided by the identification of Staphylococcus aureus by mNGS findings and the severity of the pulmonary infection. In addition, local antimicrobial surveillance data from our center have shown a relatively high rate of oxacillin/cloxacillin resistance among clinical Staphylococcus aureus isolates, making empirical coverage for methicillin-resistant strains an important consideration. Therefore, vancomycin was selected as the initial anti-staphylococcal agent. However, persistent radiographic progression and subtherapeutic trough levels suggested inadequate drug exposure. This may be attributed to altered pharmacokinetics in preterm infants, a well-recognized challenge in neonatal pharmacotherapy (6). Following transition to linezolid, the patient demonstrated gradual clinical and radiographic improvement. Given its favorable tissue penetration and bioavailability, linezolid may represent a valuable alternative in cases where vancomycin is ineffective or difficult to optimize.

In addition to pulmonary involvement, the patient developed coagulopathy and dynamic platelet changes, including initial thrombocytopenia followed by reactive thrombocytosis. These findings are consistent with previous reports of hematological abnormalities in severe varicella, which may result from bone marrow suppression, inflammatory activation, or immune-mediated mechanisms (12, 13). Furthermore, the occurrence of oral candidiasis highlights the increased risk of fungal superinfection in preterm neonates receiving prolonged broad-spectrum antibiotic therapy (14), emphasizing the need for careful monitoring.

Finally, this case raises important considerations regarding prevention. Current international guidelines recommend varicella-zoster immune globulin (VZIG) for high-risk neonates following exposure, although criteria vary (4, 6). When VZIG is unavailable, antiviral prophylaxis may serve as an alternative (15, 16). In the present case, post-exposure prophylaxis was not administered due to delayed recognition of maternal infection, representing a missed opportunity to potentially mitigate disease severity. The limited availability of VZIG in certain regions remains a significant gap in neonatal infection prevention strategies.

## Conclusions

This case highlights a rare but clinically significant presentation of neonatal varicella complicated by Staphylococcus aureus lung abscess in a preterm infant. It emphasizes that apparent resolution of cutaneous lesions does not exclude ongoing deep-seated infection, and that preterm neonates may present with atypical or subtle clinical features. Persistent respiratory abnormalities should prompt early imaging to facilitate timely diagnosis. In addition, advanced diagnostic tools such as mNGS, together with individualized antimicrobial strategies and therapeutic drug monitoring, are essential for optimizing clinical outcomes. Strengthening post-exposure prophylaxis strategies, including improving access to VZIG, may further reduce the risk of severe disease in high-risk neonates.

## Data Availability

The original contributions presented in the study are included in the article/Supplementary Materials, further inquiries can be directed to the corresponding author.

## Supplementary material

Supplementary Figure S1 Diagnostic imaging of lung abscess in neonatal varicella. (S1A) Lung ultrasound (LUS) showing irregular hypoechoic lesions with indistinct margins and heterogeneous echotexture in the right lung, with preserved A-lines and sparse B-lines. (S1B) Color Doppler imaging demonstrating internal vascular signals within the lesion. (S1C) Contrast-enhanced chest CT (venous phase, mediastinal window) on illness day 16 confirming a right upper lobe abscess measuring 21 × 27 × 23 mm.

Supplementary Figure S2 Serial chest X-ray findings showing the resolution of lung abscess secondary to neonatal varicella. (S2A) Chest X-ray on hospital day 22 (vancomycin day 13) showing limited radiological improvement. (S2B) Chest X-ray on hospital day 28 (linezolid day 6) showing marked resolution of pulmonary lesions. (S2C) Chest X-ray on illness day 53 demonstrating complete resolution of the lung abscess.
