# Supplementary figures and images for: Neonatal varicella complicated by Staphylococcus aureus lung abscess in a preterm infant: a case report
Source: Front Pediatr. 2026 Jul 6;14:1845229. doi: 10.3389/fped.2026.1845229 (PMC13381697; doi:10.3389/fped.2026.1845229)

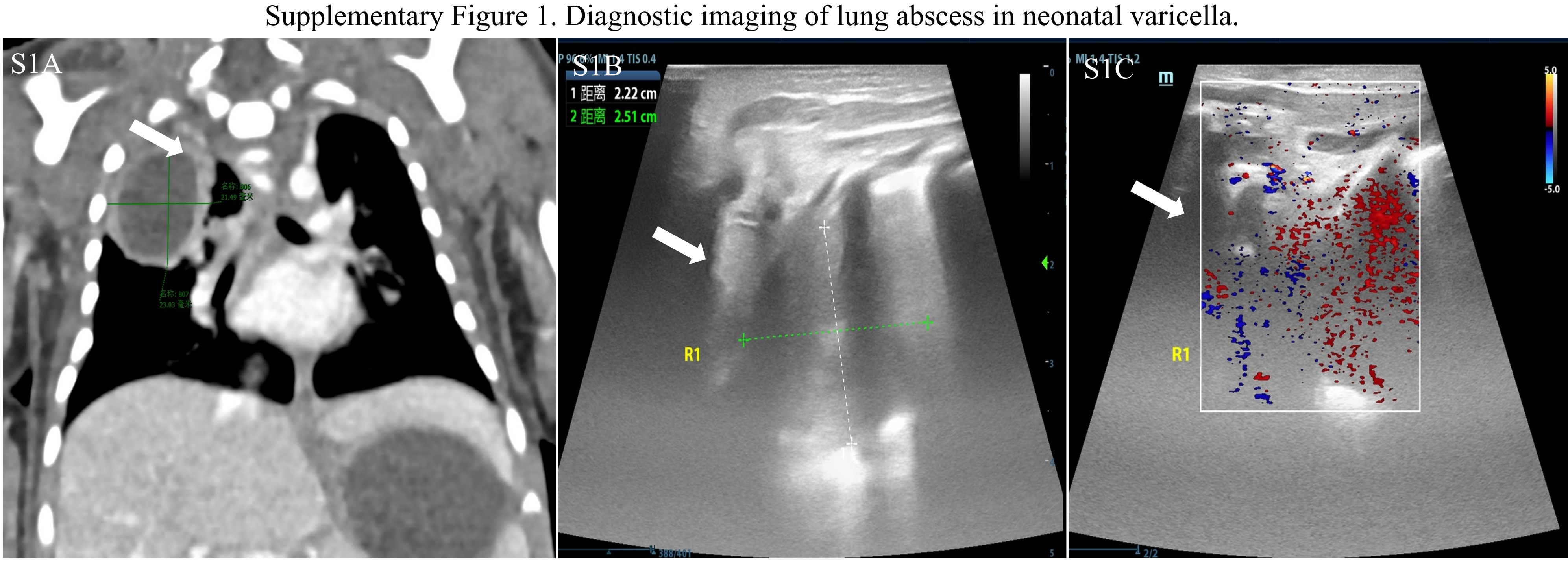

Supplement: Supplementary Figure S1 — Diagnostic imaging of lung abscess in neonatal varicella. (S1A) Lung ultrasound (LUS) showing irregular hypoechoic lesions with indistinct margins and heterogeneous echotexture in the right lung, with preserved A-lines and sparse B-lines. (S1B) Color Doppler imaging demonstrating internal vascular signals within the lesion. (S1C) Contrast-enhanced chest CT (venous phase, mediastinal window) on illness day 16 confirming a right upper lobe abscess measuring 21 × 27 × 23 mm. [file Image1.tif]

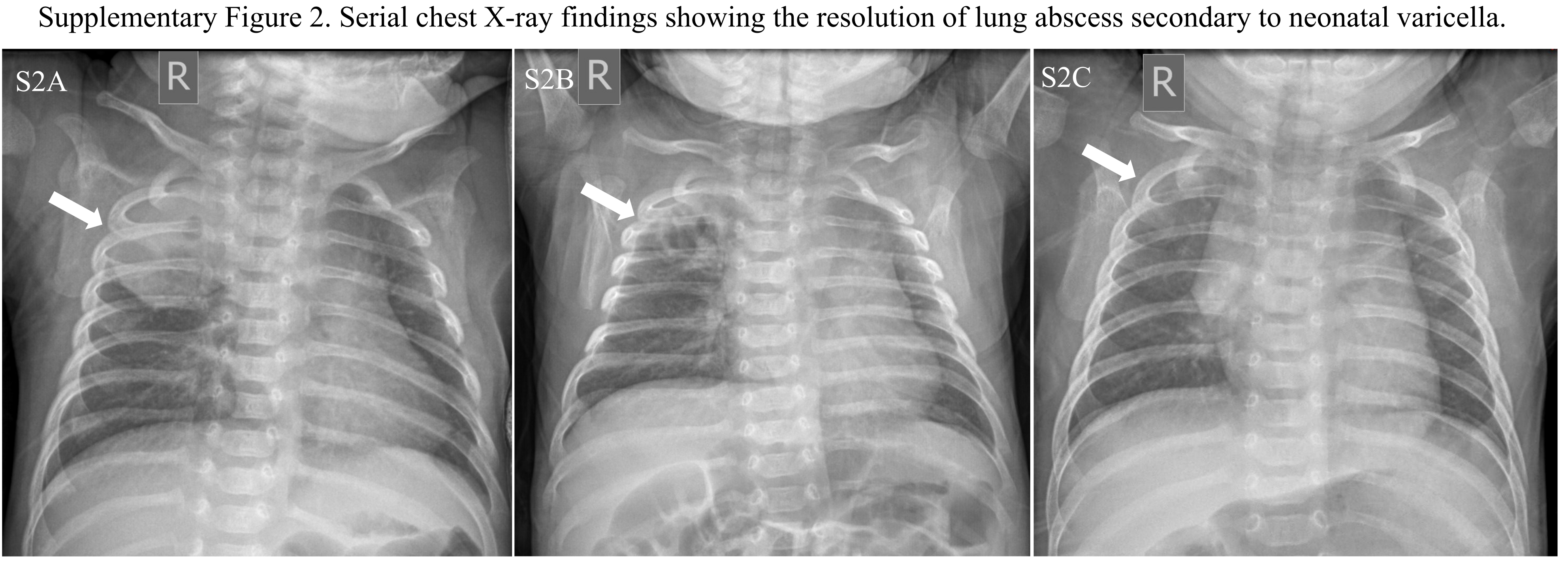

Supplement: Supplementary Figure S2 — Serial chest X-ray findings showing the resolution of lung abscess secondary to neonatal varicella. (S2A) Chest X-ray on hospital day 22 (vancomycin day 13) showing limited radiological improvement. (S2B) Chest X-ray on hospital day 28 (linezolid day 6) showing marked resolution of pulmonary lesions. (S2C) Chest X-ray on illness day 53 demonstrating complete resolution of the lung abscess. [file Image2.tif]
